# Novel immune classification based on machine learning of pathological images predicts early recurrence of hepatocellular carcinoma

**DOI:** 10.3389/fonc.2024.1391486

**Published:** 2024-05-17

**Authors:** Tianhua Tan, Huijuan Hu, Wei Zhang, Ju Cui, Zhenhua Lu, Xuefei Li, Jinghai Song

**Affiliations:** ^1^Department of General Surgery, Beijing Hospital, National Center of Gerontology, Institute of Geriatric Medicine, Chinese Academy of Medical Sciences & Peking Union Medical College, Beijing, China; ^2^Key Laboratory of Quantitative Synthetic Biology, Shenzhen Institute of Synthetic Biology, Shenzhen Institutes of Advanced Technology, Chinese Academy of Sciences, Shenzhen, Guangdong, China; ^3^Department of Pathology, Beijing Hospital, National Center of Gerontology, Institute of Geriatric Medicine, Chinese Academy of Medical Sciences, Beijing, China; ^4^The Key Laboratory of Geriatrics, Beijing Hospital, National Center of Gerontology, Institute of Geriatric Medicine, Chinese Academy of Medical Sciences, Beijing, China; ^5^Key Laboratory of Carcinogenesis and Translational Research (Ministry of Education), Department of Gastrointestinal Cancer Center, Ward I, Peking University Cancer Hospital & Institute, Beijing, China

**Keywords:** hepatocellular carcinoma, pathological images, tumor microenvironment, early recurrence, prognostic model

## Abstract

**Introduction:**

Immune infiltration within the tumor microenvironment (TME) plays a significant role in the onset and progression of hepatocellular carcinoma (HCC). Machine learning applied to pathological images offers a practical means to explore the TME at the cellular level. Our former research employed a transfer learning procedure to adapt a convolutional neural network (CNN) model for cell recognition, which could recognize tumor cells, lymphocytes, and stromal cells autonomously and accurately within the images. This study introduces a novel immune classification system based on the modified CNN model.

**Method:**

Patients with HCC from both Beijing Hospital and The Cancer Genome Atlas (TCGA) database were included in this study. Additionally, least absolute shrinkage and selection operator (LASSO) analyses, along with logistic regression, were utilized to develop a prognostic model. We proposed an immune classification based on the percentage of lymphocytes, with a threshold set at the median lymphocyte percentage.

**Result:**

Patients were categorized into high or low infiltration subtypes based on whether their lymphocyte percentages were above or below the median, respectively. Patients with different immune infiltration subtypes exhibited varying clinical features and distinct TME characteristics. The low-infiltration subtype showed a higher incidence of hypertension and fatty liver, more advanced tumor stages, downregulated immune-related genes, and higher infiltration of immunosuppressive cells. A reliable prognostic model for predicting early recurrence of HCC based on clinical features and immune classification was established. The area under the curve (AUC) of the receiver operating characteristic (ROC) curves was 0.918 and 0.814 for the training and test sets, respectively.

**Discussion:**

In conclusion, we proposed a novel immune classification system based on cell information extracted from pathological slices, provides a novel tool for prognostic evaluation in HCC.

## 1 Introduction

Liver cancer ranks as the fourth most common cause of cancer-related deaths and the sixth most frequently diagnosed cancer globally, with its highest incidence in East Asia and Africa and a rising occurrence worldwide (1). Hepatocellular carcinoma (HCC) stands as a predominant form of primary liver cancer, encompassing 75–85% of all cases (2). Patients diagnosed with early-stage HCC derive benefit from hepatic resection or transplantation, boasting a 5-year survival rate of 70%. Nonetheless, HCC exhibits a notable intrahepatic recurrence rate, with recurrence within 2 years affecting 50–70% of patients, signifying a poor prognosis (3). Recent advancements in systemic therapies have further enhanced overall survival rates (4, 5). A combination strategy of anti-angiogenesis agents with immunotherapy, bevacizumab plus atezolizumab, has been approved as the first-line treatment for patients with unresectable HCC, other anti-angiogenesis agents including regorafenib and cabozantinib have been proven to improve overall survival (OS) as second-line treatment (6). The various systemic therapies pose a new challenge for surgeons and oncologists in terms of selecting optimal personalized treatment strategies, and the study of the immune microenvironment of HCC provides evidence for addressing this challenge.

Previous studies have revealed that early recurrence of HCC is associated with both clinical and tumor traits, such as male gender, high levels of bilirubin and alpha-fetoprotein (AFP), tumor size, and microvascular invasion. Prediction models have been established based on these traits (7, 8). Advances in genomics and transcriptomics have further unveiled correlations between the tumor microenvironment (TME) and early recurrence at the molecular level (9, 10), while radiomics offers a different perspective on tumor traits (11). In addition to clinical characteristics and multiomics, pathological images also contain abundant information that has been insufficiently explored. HCC consists of a mixture of cell types, including malignant hepatocytes, immune cells, and stromal cells. Pathological images of HCC are commonly used to classify and grade tumors based on the degree of differentiation, satellite nodules, microvascular invasion, and other histological features. However, recognizing and annotating the types of individual cells in these images, and exploring the interactions among them, may provide more comprehensive information.

Lymphocytes constitute most immune cells in HCC, and studies indicate that abundant lymphocyte infiltration in HCC is associated with a better prognosis (12). Previous studies of lymphocyte infiltration primarily relied on the technique of genomics and transcriptomics, which required complicated examination and additional cost. Our previous study employed image processing techniques and adapted a convolutional neural network (CNN) initially designed for lung cancer to establish a novel cell recognition model suitable for patients with HCC (13), which classified cells autonomously and accurately in pathological images into three types: tumor cells, lymphocytes, and stromal cells (14). The cell recognition model provides a more efficient and available method to evaluate lymphocyte infiltration in the HCC landscape, reducing both time and financial cost.

Patients from the Beijing Hospital and the Liver Hepatocellular Carcinoma (LIHC) cohort in The Cancer Genome Atlas (TCGA) database were included. Given the crucial role of lymphocytes in tumor elimination and evasion, we categorized patients into high and low immune infiltration groups based on lymphocyte levels (15). Furthermore, we analyzed differences in clinical features, prognosis, and TME between these subtypes. We observed distinct disease-free survival (DFS) among different subtypes in both the Beijing Hospital and TCGA cohorts. To predict potential early recurrence of HCC (defined as DFS less than 1 year) (16), we developed a novel prognostic model based on clinical features and immune subtypes. Additionally, we created a nomogram to aid in clinical decision-making.

Our study primarily focused on individual cells within pathological images of HCC and proposed a novel immune subtype based on lymphocyte levels. These findings could offer new insights into the pathology of HCC and contribute to personalized post-operative treatment strategies.

## 2 Methods

### 2.1 Data collection and preprocessing

We examined patients who underwent surgical resection or liver transplantation between 2013 and 2019 at Beijing Hospital. Patients included in this study had to meet the following criteria: (a) be at least 18 years old; (b) have a pathological diagnosis of HCC; (c) not receive any preoperative treatment; (d) have no history of prior malignancy, autoimmune disease, or immune deficiency disease; and (e) provide well-preserved formalin-fixed paraffin-embedded (FFPE) slides with hematoxylin-eosin (H&E) staining. Patients with incomplete clinical information were excluded. Ultimately, 64 patients were included in the study.

To analyze the TME of HCC and validate the prognostic model, pathological images, clinical information, and RNA-sequencing data were retrieved from the TCGA-LIHC database via Genomic Data Commons (http://gdc.cancer.gov/). Data preprocessing was conducted to enhance the quality of data and ensure the reliability of further analysis. The gene expression RNAseq data were normalized, duplicated values and missing values were eliminated. Patients without complete survival data or pathological images were excluded. Finally, 198 patients were included.

### 2.2 Pathologic images processing pipeline

Our prior study proposed a reliable and effective pathological images processing pipeline (14). Each image was digitally captured at 40× magnification and labeled as a region of interest (ROI), defined as the major malignant region, using the ImageScope annotation tool. Subsequently, we randomly sampled 20 patches within each ROI and calculated the number of tumor cells, lymphocytes and stromal cells within these patches.

### 2.3 Immune infiltration classification

To categorize tumors into distinct immune phenotypes, we initially determined the percentage of lymphocytes and the ratio of lymphocytes to tumor cells in each image. Subsequently, we conducted a test for normality to identify the parameter with the least dispersion, selected based on the interquartile range (IQR), for further analysis (15). Patients were then stratified into two subtypes based on immune infiltration levels: high and low. This categorization was determined using the median lymphocyte percentage as the threshold. Finally, we compared clinical features and prognosis between these two subtypes.

### 2.4 Functional enrichment analysis

We identified differentially expressed genes (DEGs) among various subtypes using the DESeq2 R package, employing criteria of a base mean > 10, log2 Fold Change > 1, and adjusted P value < 0.05 (17). Subsequently, we conducted Gene Ontology (GO) functional pathway enrichment analysis using the clusterProfiler R package, with significance determined at a P value < 0.05 (18). Furthermore, we obtained HALLMARK- and KEGG-related gene datasets from the Gene Set Enrichment Analysis (GSEA) official website. We then performed GSEA utilizing the GSEA algorithm (19) and Gene Set Variation Analysis (GSVA) employing the GSVA R package (20).

### 2.5 Evaluation of immune features

The ESTIMATE (Estimation of STromal and Immune cells in Malignant Tumor tissues using expression data) analysis was performed to assess the level of immune infiltration, utilizing the estimate R package (21). Additionally, Cell type Identification By Estimating Relative Subsets Of RNA Transcripts (CIBERSORT) analysis was employed to determine the relative abundance of 22 different immune cell types within the tumor tissue (22). Furthermore, Tumor Immune Dysfunction and Exclusion (TIDE) analysis was carried out to evaluate the potential for tumor immune escape, utilizing the TIDE website (http://tide.dfci.harvard.edu/) (23).

### 2.6 Prognostic model establishment and validation

To further investigate the prognostic value of immune classification, we categorized patients into two groups: a good prognosis group and a poor prognosis group, defined as having a DFS > 1 year (16). From the Beijing Hospital cohort, we collected 55 variables comprising clinical and pathological features. We then developed Receiver Operating Characteristic (ROC) curves for each variable using the pROC R package and extracted the Area Under the Curve (AUC) for evaluation (24). Variables with an AUC exceeding 0.6 were selected, and Least Absolute Shrinkage and Selection Operator (LASSO) analysis was employed to reduce the number of variables in the risk model using the Glmnet R package (25). A 20-fold cross-validation was conducted to identify the optimal lambda value. “Lambda.1se” was utilized to determine the minimum number of independent variables required for a well-performing model. Subsequently, we employed the Beijing Hospital cohort as the training set and 58 patients from the TCGA-LIHC database, who provided complete clinical information, as the test set. Logistic regression was then applied to establish a prognostic model, and ROC curves were generated for both the training and test sets. Finally, a nomogram was constructed based on the prognostic model.

### 2.7 Statistical analysis

OS was defined as the period between the day of pathological diagnosis and the day of death, while DFS was defined as the duration between the day of pathological diagnosis and the occurrence of tumor recurrence, metastasis, or death. Patients who remained free of recurrence were censored at the final follow-up. Survival analysis was conducted using Kaplan–Meier (K-M) analysis employing the Survival and Survminer R packages. Categorical and non-normally distributed measurement variables were compared using the Wilcoxon test, whereas normally distributed measurement variables were compared using the t-test. All statistical analyses were performed using R software (version 4.1.3).

## 3 Results

### 3.1 Cell type recognition and immune subtype classification

Our prior research had developed an adapted CNN model to recognize cells autonomously and accurately in pathological images of patients with HCC, with classification accuracies of 95.7%, 92.3%, and 77.6% for tumor cells, lymphocytes, and stromal cells, respectively (14).

**Figure 1:**
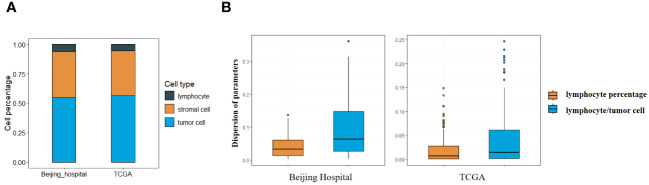
**(A)** Percentage of different types of cells in Beijing Hospital and TCGA cohorts. **(B)** Central tendency for lymphocyte percentage and lymphocyte/tumor cell ratio.

The adapted CNN model was utilized in this study, applied to both the Beijing Hospital and the TCGA cohort. Analysis of cell type percentages revealed no significant disparities between the two cohorts (**Figure 1A**). Lymphocyts, stromal cells and tumor cells account for 6.26%, 38.76% and 70.52% in the Beijing Hospital cohort, and 5.56%, 37.70% and 66.44% in the TCGA cohort respectively.

Lymphocyte percentage and lymphocyte/tumor cell ratio were computed as potential parameters for further analyses, the parameter with a lower degree of dispersion serves as the basis for subsequent grouping. Both parameters constituted non-normally distributed continuous variables, with the dispersion of lymphocyte percentage being less pronounced (IQR 0.07 vs. 0.14 in the Beijing cohort, 0.04 vs. 0.09 in the TCGA cohort, as depicted in **Figure 1B**), so that the lymphocyte percentage was selected as the parameter for stratification (26). Images with lymphocyte percentages above or below the median were categorized as having high or low immune infiltration (median = 0.039 in the Beijing cohort, median = 0.011 in the TCGA cohort). Lymphocyte percentages falling 1.5 times below Q1 or exceeding 1.5 times above Q3 were identified as outliers, 2 outliers in Beijing cohort and 14 outliers in TCGA cohort were excluded.

### 3.2 Patients in different immune subtypes presented variant clinical features and prognosis

We categorized the patients into high- and low-immune cell infiltration subtypes based on the threshold described above. For patients in the Beijing Hospital cohort, we collected data on 17 parameters, including epidemiological factors, indicators of liver function, medical history, tumor stage, and pathohistological features of the tumor, and compared the two subtypes (refer to **Table 1**). The low-infiltration subtype exhibited a higher incidence of hypertension (51.4% vs. 17.2%, p = 0.010) and fatty liver (25.7% vs. 3.4%, p = 0.017), and displayed a larger tumor diameter (median 5.0 vs 4.5, p = 0.048). Additionally, the low-infiltration subtype demonstrated a higher incidence of satellite nodules, elevated AFP levels, and a more advanced TNM stage, but showed no statistical significance. These findings suggest that lower immune infiltration may be associated with a history of metabolic syndrome and may promote tumor progression.

**Table 1:** Clinical and pathological characteristics of patients in the 2 subtypes*.

Characteristic	Low infiltration (n = 35)	High infiltration (n = 29)	P value
Male	29 (82.8)	23 (79.3)	0.968
Age, Mean ± SD, **years**	61.94 ± 14.97	57.17 ± 12.21	0.165
Alb*, Median (Q1,Q3), **g/L**	40 (39.5, 41)	41 (40, 43)	0.101
TB*, Median (Q1,Q3), **μmol/L**	12 (8.7, 15.15)	11.5 (9.3, 14.1)	0.914
PT*, Mean ± SD, **s**	11.47 ± 1.11	11.39 ± 0.94	0.770
AFP* (≥400 ng/ml)	12 (34.2)	3 (10.3)	0.051
Diabetes	11 (31.4)	3 (10.3)	0.084
Hypertension	18 (51.4)	5 (17.2)	0.010
Alcohol	11 (31.4)	8 (27.6)	0.952
Hepatitis	26 (74.3)	24 (82.8)	0.608
Liver cirrhosis	24 (68.6)	21 (72.4)	0.952
Fatty liver	9 (25.7)	1 (3.4)	0.017
MVI*			0.389
M0	20 (57.1)	20 (69.0)	
M1	10 (28.6)	4 (13.8)	
M2	5 (14.3)	5 (17.2)	
Tumor size, Median (Q1,Q3), **cm**	5.0 (3.5, 9.5)	4.5 (2.5, 7.0)	0.048
Vascular invasion, n (%)	8 (22.9)	4 (13.8)	0.546
Satellite nodules, n (%)	12 (34.3)	3 (10.3)	0.051
TNM Stage, n (%)			0.050
Stage 1	16 (45.7)	20 (69.0)	
Stage 2	15 (42.9)	5 (17.2)	
Stage 3	2 (5.7)	4 (13.8)	
Stage 4	2 (5.7)	0 (0)	

*Values are numbers of patients (percentages) unless otherwise indicated; Alb for albumin; TB for total bilirubin; PT for prothrombin time; AFP for alpha-fetoprotein; MVI for microvascular invasion, M0 means no MVI, M1 means less than 5 MVI occurred within 1 cm from the tumor, M2 means more than 5 MVI or MVI occurred 1 cm away from the tumor.

**Figure 2:**
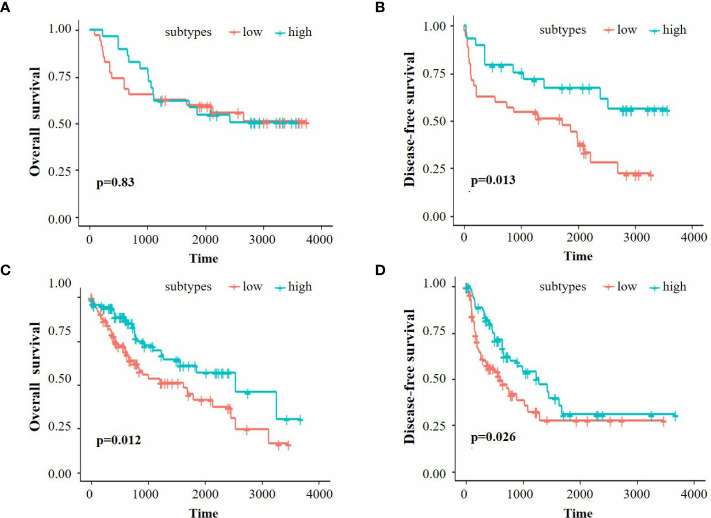
**(A, B)** K-M survival analysis of OS and DFS for the Beijing Hospital cohort. **(C, D)** K-M survival analysis of OS and DFS for the TCGA cohort.

We further performed K-M analysis on both the Beijing Hospital and TCGA cohorts to assess the prognostic value of immune classification. In the Beijing Hospital cohort, patients with the high-infiltration subtype exhibited a favorable prognosis in terms of DFS (p=0.013), but no significant difference was observed in OS (**Figures 2A, B**). In the TCGA cohort, the high infiltration subtype demonstrated a favorable prognosis in both OS and DFS (**Figures 2C, D**, p=0.012 for OS, p=0.026 for DFS).

### 3.3 Different immune subtypes present a distinct TME

**Figure 3:**
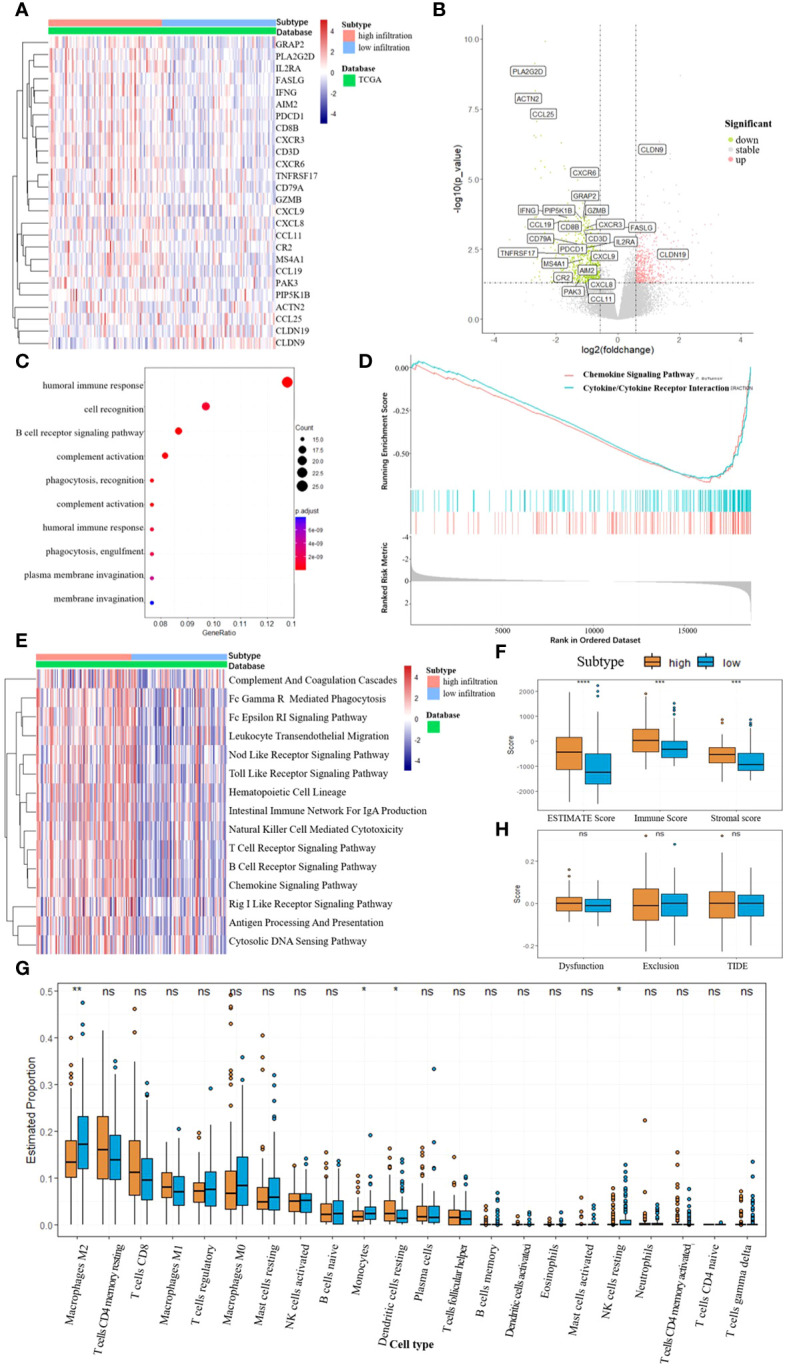
**(A, B)** Differentially expressed genes between the two subtypes, DEGs related to immune pathways were annotated. **(C)** GO enrichment, biological process (BP). **(D)** Top 2 pathway enriched in GSEA analysis. **(E)** GSVA enrichment according to the KEGG database. **(F)** ESTIMATE scores between the two subtypes, *** p<0.001, **** p<0.0001. **(G)** Abundance of different immune cells between the two subtypes, * p<0.05, ** p<0.01, NS for not significant. **(H)** TIDE scores between the two subtypes, NS for not significant.

RNA sequencing data were gathered from the TCGA-LIHC database. We identified the DEGs between the two subtypes using the DESeq2 R package and annotated genes associated with immune pathways according to the KEGG database (**Figures 3A, B**). The analysis revealed that most of the immune-related genes were down-regulated in the low infiltration subtype. To delve deeper into the discrepancies in cellular function between the subtypes, we performed functional enrichment analyses utilizing the GO, GSEA, and GSVA methodologies (**Figures 3C, D, E**). The top 10 pathways enriched in the GO analysis (sorted by qvaule, increased) were all linked to immune function. Meanwhile, the top two pathways enriched in the GSEA analysis (ranked by absolute NES, decreased) were the chemokine and cytokine signaling pathways. The extent of immune infiltration was quantified using ESTIMATE analysis, and the estimated immune and stromal scores were compared between the two subtypes using the Wilcoxon test (**Figure 3F**). The high-infiltration subtype exhibited higher scores, indicating a greater degree of immune cell infiltration in the TME. Subsequently, CIBERSORT analysis was performed to assess immune cell abundance in the two subtypes (**Figure 3G**). The findings revealed that the low-infiltration subtype manifested a higher level of type 2 macrophages (M2), monocytes, and resting natural killer (NK) cells, suggesting a propensity towards immune suppression. Finally, TIDE analysis was employed to evaluate the potential for tumor escape, indicating no significant difference between the two subtypes (**Figure 3H**).

### 3.4 Establishment and validation of the prognostic model based on immune subtypes and clinical features

**Figure 4:**
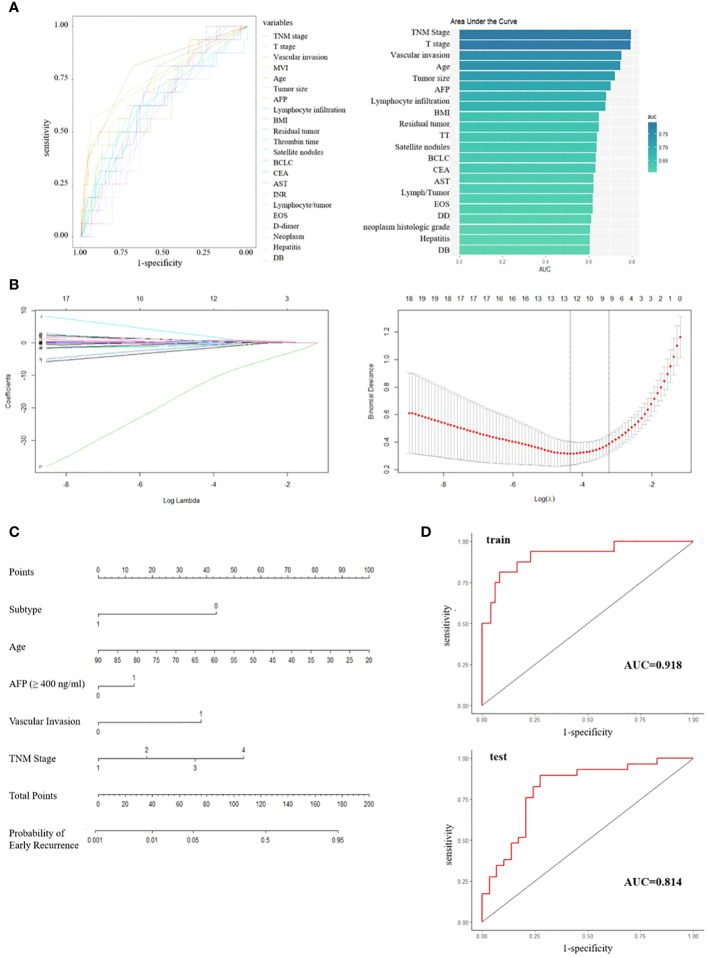
**(A)** ROC curves and ranked AUC of clinical and pathological features. **(B)** LASSO regression and cross-validation for variable selection. **(C)** Nomogram for predicting early recurrence; Subtype, 0/1 means low/high infiltration subtype; AFP, 0/1 means the level of AFP less than 400 ng/ml or not; Vascular invasion, 0/1 means no/any type of vascular invasion; TNM stage, 1/2/3/4 means stage I/II/III/IV respectively. **(D)** ROC curves for training set and test set.

Patients with a DFS shorter or longer than 1 year were classified into poor or good prognosis groups. A total of 55 variables, including clinical and pathological features, were collected from the patients in the Beijing Hospital cohort. ROC curves were developed for each variable to evaluate their predictive value, and variables with an AUC greater than 0.6 were listed (**Figure 4A**). The immune classification ranked 8 (AUC = 0.67), while other variables with high rankings were mostly tumor features, such as TNM stage, microvascular invasion (MVI), and serum AFP level, which were consistent with previous findings (7, 8). To develop a prognostic model for patient outcomes, we used all patients from the Beijing Hospital cohort as the training set and 58 patients from the TCGA cohort with complete clinical information as the test set. We performed LASSO analysis and cross-validation to reduce the number of variables and determine the minimum number of variables needed for a model with favorable performance (**Figure 4B**). Finally, five variables were included in the logistic regression analysis: immune classification, age, AFP level, vascular invasion, and TNM stage, and a nomogram was developed (**Figure 4C**). ROC curves were generated for both the training and test sets, with AUCs of 0.918 and 0.814, respectively (**Figure 4D**).

## 4 Discussion

Benefiting from advanced genomics and transcriptomics technologies, the TME of HCC has been extensively explored in recent years. This exploration has revealed impressive immune heterogeneity, fueling the development of immune therapies for HCC, such as PD1/PDL1 inhibitors. Apart from immune heterogeneity, spatial heterogeneity also significantly influences tumor progression and metastasis (27). While studies on spatial heterogeneity have primarily focused on the gene or molecular level using techniques like single-cell RNA sequencing and spatial transcriptomics (28, 29). Examining the spatial distribution of different cell types within the TME could offer a novel perspective.

Pathological images serve as the gold standard for tumor diagnosis, containing vast amounts of information that warrant further investigation. Traditional pathological research methods, such as immunohistochemistry and fluorescence *in situ* hybridization, operate at the molecular level and often require additional experiments. Hence, a method that directly extracts cellular information from H&E-stained pathological images could prove more efficient. The primary challenge lies in accurately and efficiently recognizing and classifying cells within these images. Previous studies on lung cancer have successfully developed reliable deep-learning models capable of identifying different cell types in pathological images of lung adenocarcinoma and non-small cell lung cancer (30–32). However, such models have seen limited application in HCC (33). Our previous study proposed an effective pathological image processing pipeline and adapted a CNN model to classify the cells in the pathological images of patients with HCC.

Based on the modified CNN model, we have proposed a novel immune classification based on the percentage of lymphocytes in the images. We hypothesize that this novel immune classification holds potential prognostic value. Upon dividing patients into high and low immune infiltration groups, we observed that the low-infiltration subtype exhibited a higher incidence of hypertension and fatty liver. This suggests that metabolic disturbances may impact immune infiltration in the TME. Further analysis of RNA sequencing data from the TCGA dataset confirmed the reasonability and reliability of our novel immune classification system. The next objective of our study was to establish a prognostic model based on this novel immune classification. We utilized the Beijing Hospital cohort as the training set and the TCGA cohort as the test set. Patients were divided into poor/good prognosis groups according to DFS. We conducted LASSO analysis and logistic regression on 55 variables and developed a nomogram for prognosis prediction. The AUC of the ROC curves was 0.918 and 0.814 for the training set and the test set, respectively. The variables included in the nomogram were immune classification, age, AFP level, TNM stage, and vascular invasion status. Except for immune classification, all other variables were available in the process of HCC treatment. Our modified CNN model also facilitated the determination of immune classification. With this nomogram, we can conveniently evaluate the risk of early recurrence in patients diagnosed with HCC who undergo surgical resection or liver transplantation. For patients at high risk of early recurrence, more intensive follow-up and a more proactive postoperative treatment strategy are warranted.

This study is a single-center retrospective study, and only 64 patients were included in the Beijing Hospital cohort, which inevitably limits the reliability of its results and the prognostic value of the proposed model. The utilization of lymphocyte percentage as the sole parameter for immune classification appears insufficient. To address these limitations, a multi-center prospective study design is necessary, along with more comprehensive investigations exploring the spatial relationships among various cell types. Furthermore, the predictive value of our novel immune classification in response to various immunotherapy strategies merits further exploration. Our future research efforts will be focused on addressing these challenges.

An unexpected finding of this study was the observation that patients with different immune infiltration subtypes exhibited distinct histories of metabolic syndrome. This discovery underscores the importance of investigating the correlation and interaction between metabolic and immune pathways in the TME, a topic that warrants further exploration.

Overall, our study proposed a novel immune classification system based on a reliable cell recognition model and demonstrated favorable prognostic value. The novel prognostic model and nomogram, developed from clinical features and immune classification, could serve as practical tools for evaluating the risk of early recurrence in patients with HCC. Moreover, they could provide reliable suggestions for postoperative clinical decision-making.

## Data availability statement

The raw data supporting the conclusions of this article will be made available by the authors, without undue reservation.

## Ethics statement

The studies involving humans were approved by Ethics committee, Beijing Hospital. The studies were conducted in accordance with the local legislation and institutional requirements. The human samples used in this study were acquired from a by- product of routine care or industry. Written informed consent for participation was not required from the participants or the participants’ legal guardians/next of kin in accordance with the national legislation and institutional requirements.

## Author contributions

TT: Data curation, Formal analysis, Investigation, Methodology, Project administration, Visualization, Writing – original draft, Writing – review & editing. HH: Investigation, Methodology, Project administration, Resources, Software, Writing – review & editing. WZ: Funding acquisition, Writing – review & editing. JC: Conceptualization, Data curation, Methodology, Supervision, Validation, Writing – review & editing. ZL: Writing – review & editing. XL: Conceptualization, Supervision, Validation, Writing – review & editing. JS: Conceptualization, Funding acquisition, Resources, Supervision, Validation, Writing – review & editing.
